# Prevalence and factors associated with suicidal ideation amongst college students in the Nelson Mandela Bay Municipality, South Africa

**DOI:** 10.4102/safp.v63i1.5195

**Published:** 2021-01-29

**Authors:** Adeyinka A. Alabi, Olawumi K. Oladimeji, Oladele V. Adeniyi

**Affiliations:** 1 Department of Family Medicine, Walter Sisulu University, Port Elizabeth, South Africa; 2 Department of Family Medicine, Dora Nginza Provincial Hospital, Port Elizabeth, South Africa; 3 Eastcape Midlands TVET College, Uitenhage, South Africa; 4 Department of Family Medicine, Walter Sisulu University, East London, South Africa; 5 Department of Family Medicine, Cecilia Makiwane Hospital, East London, South Africa

**Keywords:** suicidal behaviour, suicidal plans, higher education, students, South Africa

## Abstract

**Background:**

Suicidal behaviour amongst college students constitutes a significant social and public health problem globally. This study determined the prevalence and associated factors of suicidal ideation amongst students of higher education in the Nelson Mandela Bay Municipality (NMBM), South Africa.

**Methods:**

In this institution-based cross-sectional study, a multistage cluster sampling of 826 participants, drawn from a college in NMBM, was conducted from January to March 2020. Data were collected with a standardised self-administered questionnaire. Multivariable logistic regression analysis was used to identify the factors associated with suicidal ideation.

**Results:**

Participants’ ages ranged from 18 to 24 years, with a mean age of 20.49 years (standard deviation, 1.88 years). The lifetime prevalence of suicidal ideation and plans in the preceding 12 months were 24.5% and 9.6%, respectively. The odds of suicidal ideation were higher in students who experienced bullying (adjusted odds ratio [AOR], 1.89; 95% confidence interval [CI], 1.35–2.65), mental illness (AOR, 1.89; 95% CI, 1.35–2.65), a history of sexual assault (AOR, 2.50; 95% CI, 1.20–5.21) and experience of sexual assault by or to a close family member (AOR, 1.69; 95% CI, 1.01–2.82). Underlying chronic illness was associated with a twofold risk for suicidal ideation in both sexes.

**Conclusion:**

About a quarter of the students sampled at the college had experienced suicidal ideation and some had had suicidal plans in the preceding 12 months. Screening for the identified risk factors amongst the student population coupled with prompt interventions would mitigate the risk of suicide in the study population.

## Introduction

Suicide constitutes a significant social and public problem globally, with one person dying by suicide every 40 s and many more people attempting suicide.^1^ Worldwide, suicide constitutes the second leading cause of death amongst people between the ages of 15 years and 29 years.^2^ It is also the second leading cause of death amongst college students in the United States of America.^3^ In South Africa, suicide is responsible for one out of every 10 unnatural deaths amongst young people, and many more engage in deliberate self-harm annually.^4^ There appears to be an increasing trend towards suicidal behaviour in the past 15 years, and this calls for preventative intervention.^5^

Suicidal behaviour encompasses suicidal ideation, deliberate self-harm and completed suicide.^6^ Suicidal ideation has been identified as an important precursor of completed suicides, and young people with suicidal ideation have a higher risk of committing suicide than those without ideation.^7,8^ Similarly, Montier et al. (2019) reported that about half of students with suicidal ideation transitioned to suicidal plans, whilst one-quarter progressed to suicide attempts.^9^ Therefore, timely identification of modifiable and non-modifiable risk factors for suicidal ideation should be part of intervention strategies to reduce deaths by suicide. With the World Health Organization’s (WHO) 2020 deadline of the envisioned target of a 10% reduction in suicide approaching,^10^ a clear understanding of the risk factors for suicidal ideation would assist in crafting institution-specific preventative strategies.

Several studies conducted in both low- and high-income countries have reported an association between environmental, psychosocial, biological and behavioural factors and suicidal ideation amongst college students.^11,12^ Worldwide, mental illnesses such as depression, anxiety, personality disorders and bipolar disorders seem to be consistent risk factors for suicidal ideation amongst college students.^13,14^ College students are vulnerable to mental illness because of the stress of separation from their family support system, challenging academic load, financial constraint and peer pressure; such mental illnesses put them more at risk of suicidal ideation.^15,16^

In addition to primary mental illness, psychological trauma such as bullying, sexual assault, intimate partner violence and childhood adverse events appear to have a significant association with suicidal ideation.^17,18,19,20^ Previous studies have shown that a poor level of financial support, unemployment of parents and family instability are risk factors for suicidal ideation amongst students.^21,22^ Because of the dearth of published studies on suicidal ideation amongst students at institutions of higher learning in South Africa, it is unclear what the prevalence and risk factors for suicidal ideation are amongst such students in the country. Even more uncertain is the proportion of these students who transitioned to developing plans to commit suicide in the past year. These findings will assist higher education institutions’ (HEIs) administrators in crafting appropriate interventions to mitigate the risk of suicide amongst the student population. This study determines the prevalence of suicidal ideation and plans amongst students of higher education in the Nelson Mandela Bay Municipality (NMBM), South Africa. In addition, the study further examines the factors associated with suicidal ideation in the cohort.

## Methods

### Study design and setting

This institution-based cross-sectional study was conducted between 15 January and 15 March 2020 at the Eastcape Midlands College, situated in NMBM, South Africa. Eastcape Midlands College, located in Uitenhage, has seven campuses and offers courses in Engineering, Business and occupational training programmes. A total number of 8563 students were registered for the 2019/2020 study sessions at all campuses. The distribution of students was as follows: Charles Goodyear Campus, 1308; High Street Campus, 673; Thanduxolo Campus, 904; Brickfields Road Campus, 416; Graaff-Reinet Campus, 807; Grahamstown Campus, 1677; Heath Park Campus, 1336; and Park Avenue Campus, 1442.

### Study population and sample size estimation

All students in the various programmes and stated year of study at the Eastcape Midlands College in NMBM were eligible for the study. Participants were included if they had registered for the 2019/2020 academic year, were at least 18 years old at the time of the study and were willing to complete a 15-min questionnaire. However, participants who were clinically unwell were excluded from the study. In order to ensure that the findings would be generalisable to the entire student population, a sample size of 902 was calculated by using the formula for cross-sectional studies:

$$\text{N}=\text{PQ}\times 4/0.2^{2}$$ [Eqn 1]

where P is the expected prevalence of students with suicidal ideation (P was set as 9.1% as reported by a South African study^23^ for an error risk of 0.05), and Q = 1 – P. An initial total of 827 participants was calculated; then an additional 75 participants were added to bring the total to 902, in anticipation of incomplete responses on the main outcome measure (suicidal ideation and plans) from the students.

### Sampling technique

The study adopted a multistage cluster random sampling technique in selecting the participants. Out of seven campuses, we randomly selected four campuses (Thanduxolo, Charles Goodyear, Heath Park and Park Avenue), and each campus was clustered into lecture rooms/classes. For inclusiveness, participants were stratified based on the years and campuses of study. The second sampling involved random selection of classes from the clusters at each of the selected campus, and all the students who were present in the selected classes were invited to participate in this study voluntarily. Recruitment of participants corresponded with the sizes of clusters, campus and year of study. Four trained research assistants distributed the questionnaire to be self-administered.

### Intervention for participants

In line with the principle of beneficence, participants who self-reported suicidal ideation and plans were referred for full psychological assessment at the nearest hospital in the NMBM or at any private institution of their choice. Furthermore, the research assistants had debriefing sessions during the course of the study.

### Measures

#### Outcome variable

The main outcome for this study was the occurrence of suicidal ideation and plans. Suicidal ideation was measured by asking the participants if they had ever experienced suicidal thoughts in their lives. Furthermore, suicidal plans were assessed by asking the participants if they had planned to kill themselves in the preceding 12 months. Binary responses of ‘yes’ or ‘no’ were coded as 1 = yes and 2 = no.

#### Independent variables

The independent variables were selected based on the existing literature. They were divided into sections, where the first one captured the demographic characteristics of the participants (age, gender, primary language and year of study). Section 2 comprised questions that explored the potential drivers of suicidal ideation: socio-economic, clinical factors (chronic illnesses, substance use) and academic status. Section 3 explored the predisposing factors to suicidal ideation and planning. The experience of being bullied was assessed by asking, ‘Have you ever been bullied by another student in any manner?’ Economic status was assessed by obtaining information about the employment status of parents, total monthly family income and the number of household members. Bursary and other student loans were excluded because most of the college students were bursary recipients. The socio-economic status of the participants was assessed by the occupational status of one or both parents, the total household income and the number of household members, the self-rating of financial support and the rating of meeting of financial obligations by the participants.

Participants were also asked to rate their perceptions of their financial support from being very poor to very good. Relationship status and experience of abuse were also explored with rating questions. The presence of chronic illnesses was explored through a number of yes/no questions (yes = 1, no = 2). Relationship status with a partner and satisfaction were assessed and coded. Perception of body image was evaluated by asking the participant, ‘How would you describe your body weight?’ The expected responses included ‘underweight’, ‘normal weight’, ‘overweight’ or ‘obese’. Participants’ perception of their physical appearance was also assessed by asking, ‘How do you feel about your physical appearance?’ Probable responses included ‘I feel perfect’, ‘moderately good’ or ‘bad’.

Section 4 explored suicidal ideation and plans with four questions: (1) ‘In the past month, did you think that you would be better off dead or wish you were dead?’ (2) ‘In the past 12 months, did you think about harming yourself with the intention of killing yourself?’ (3) ‘In the past 12 months, did you ever seriously think of taking your life?’ (4) ‘In the past 12 months, have you made any plan to kill yourself?’ Responses were coded as ‘yes’ = 1 or ‘no’ = 2. Suicidal ideation was defined as responding ‘yes’ to any of the questions. Chronic illness was assessed by asking the participants if they had been diagnosed by a health professional with any of the following chronic illnesses: chronic pain, human immunodeficiency virus (HIV), depression, anxiety, bipolar disorder, schizophrenia or unknown chronic illness. Their responses were coded as 1 for ‘no’ and 2 for ‘yes’.

In order to ascertain the validity of the questionnaire, a pilot study was conducted with 10 students from one of the schools. Minor adjustments were made after reviewing the responses as well as feedback from the participants in the pilot study. The results of the pilot study were not included in the main study.

### Data analysis

The responses of participants were coded and entered into Epi Info version 3.5.3 software. Data analysis was performed using STATA software version 15.0 (Stata Corporation, College Station, TX, US). Descriptive analyses (frequencies, percentage and standard deviations) were used to describe the sociodemographic characteristics and to estimate the prevalence of suicidal ideation and planning. The chi-square test and bivariate analysis were used to assess the associations between suicidal ideation and independent variables. Multiple logistic regression analysis was fitted to assess the effect of independent variables on suicidal ideation, after adjusting for probable confounding variables. Variables that were significant at the *p* < 0.05 level were reported for factors associated with suicidal ideation. The associations were measured using odds ratios (ORs) and 95% confidence intervals (95% CIs).

### Ethical consideration

Ethical approval was obtained from the Walter Sisulu University’s Ethics Committee (reference no. 038/2019), and permission was obtained from the Department of Health (reference no. EC_201912005). In addition, permission was obtained from the management of the selected campuses prior to the implementation of the study protocol. The participants gave written informed consent demonstrating their willingness to voluntarily participate in the study. There was no financial inducement for participation. Furthermore, each participant received an information leaflet detailing the purpose and process of the study. The right to privacy and confidentiality of medical information was respected during and after the study. All research processes followed the Helsinki Declaration and laid out ethical procedures.

## Results

A total of 902 questionnaires were administered, but only 855 participants returned the questionnaires, thus giving a response rate of 94.8%. Of the returned questionnaires, 29 were incomplete and therefore were excluded from the final analysis, owing to missing information about suicidal ideation. Of these, 19 were female responses and 10 were those of males.

### Demographic characteristics of the participants

A total sample of 826 was included in the final analysis, 509 women (61.6%) and 317 men (38.4%). Overall, Charles Goodyear Campus had 323 respondents (37.8%), High Street Campus had 242 respondents (28.3%), Thanduxolo Campus had 137 respondents (16%), Park Avenue Campus had 135 respondents (15.8%) and 18 respondents (2.1%) did not indicate their campus. Furthermore, the largest number of respondents (382) were in their first year of study (44.7%), followed by year 2 (*n* = 286; 33.4%) and lastly, year 3 (*n* = 187; 21.9%).

Table 1 displays the descriptive statistics of the participants. The ages of the participants ranged from 18 to 24 years with a mean age of 20.49 years (SD, 1.88). The majority of the participants were from urban communities (70.6%), had never used cannabis (79.2%), reported having used alcohol (74.4%) and had no underlying chronic illnesses (75.2%). Chronic pain, HIV, depression, anxiety, bipolar disorder and schizophrenia were reported by 10.9%, 3.5%, 11.0%, 6.4%, 1.8% and 0.2% of the sample, respectively (Table 1).

**TABLE 1:** Background characteristics of respondents stratified by sex.

Variables	Male		Female		All participants	
	*n*	%	*n*	%	*n*	%
**All respondents**	317	38.4	509	61.6	826	100.0
**Family structure**						
Single parent	130	41.0	240	47.2	370	44.7
Both parents	149	47.0	204	40.1	353	42.7
Foster parents	38	12.0	65	12.8	103	12.5
**Parents’ occupation**						
Both employed	68	21.5	81	15.9	149	18
One employed	124	39.1	220	43.2	344	41.6
Non-employed	125	39.4	208	40.9	333	40.3
**Place of residence**						
Rural	102	32.2	142	27.9	244	29.5
Urban	215	67.8	367	72.1	582	70.5
**Rate family financial support**						
Very poor	21	6.6	48	9.4	69	8.4
Poor	44	13.9	98	19.3	142	17.2
Moderate	140	44.2	182	35.8	322	39
Good	77	24.3	114	22.4	191	23.1
Very good	35	11.0	67	13.2	102	12.3
**Have all needs met**						
Yes	144	45.4	227	44.6	371	44.9
No	173	54.6	282	55.4	455	55.1
**Alcohol use**						
Used alcohol in the last 30 days	164	51.7	234	46	398	48.2
Used alcohol before the last 30 days	74	23.3	142	27.9	216	26.2
Never used alcohol	79	24.9	133	26.1	212	25.7
**Use cannabis**						
Yes	93	29.3	79	15.5	172	20.8
No	224	70.7	430	84.5	654	79.2
**Have underlying medical conditions**						
Yes	66	20.8	139	27.3	205	24.8
No	251	79.2	370	72.7	621	75.2
**Experienced education failures**						
Yes	106	39.8	160	31.4	266	32.2
No	211	66.6	349	68.6	560	67.8
**Experienced sexual abuse**						
I am a survivor	11	3.5	42	8.3	53	6.4
Someone close is a survivor	27	8.5	98	19.3	125	15.1
I have never experienced sexual abuse	279	88.0	369	56.9	648	78.5
**Experienced unintended pregnancy**						
Yes	24	7.6	57	11.2	81	9.8
No	293	92.4	452	88.8	45	90.2
**Conflictual relationship**						
Yes	69	21.8	141	27.7	210	25.4
No	248	78.2	368	72.3	616	74.6
**Body weight perceptions**						
Normal	246	77.6	367	71.1	613	74.2
Abnormal	71	22.4	142	27.9	213	25.8
**Feel your life is better off than friends**						
Yes	150	47.3	202	39.7	352	42.6
No	167	52.7	307	60.3	474	57.4

More participants were from single-parent families (44.8%) compared to two-parent families (42.7%). More female participants (47.2%) came from single-parent homes in comparison to male participants (41.0%). Only 27.2% of the participants perceived that their financial needs were met by the family. Overall, 32.2% of the participants had experienced educational failures. The proportion of participants, who reported conflictual relationships, unwanted pregnancy and sexual abuse, was 25.4%, 9.8% and 6.4%, respectively. About a quarter of the participants perceived that they had abnormal body weight, and more than half of all the participants felt that they were not better off than their friends.

### Prevalence of suicidal ideations and plan

**FIGURE 1:**
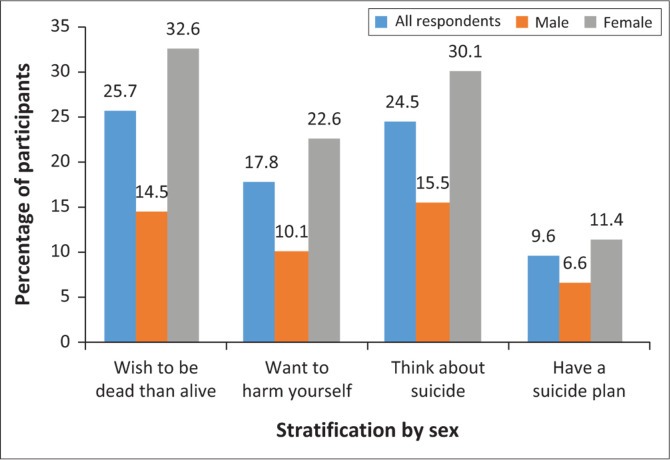
Proportion of respondents having suicidal ideation stratified by sex.

The lifetime prevalence of suicidal ideation was 24.5%, with significant gender variation (Figure 1). Significantly, more women than men reported lifetime suicidal ideation (30.1% vs. 15.5%). Amongst all the participants with suicidal ideations (*n* = 79), 9.6% had planned to commit suicide in the preceding 12 months. The lifetime prevalence of suicidal plan was 11.4% amongst women and 6.6% amongst men.

### Factors associated with suicidal ideation (multiple logistic regression analysis)

In the crude and adjusted multiple logistic model analysis (Table 2), male sex, feeling better off financially than friends, moderate or perfect feeling about body appearance, and having all needs met were associated with lower odds of experiencing suicidal ideation. The magnitude and direction of association between these factors and suicidal ideation remained after adjusting for confounding variables, except for having all needs met.

**TABLE 2:** Multiple logistic regression model analysis showing factors associated with suicidal ideation.

Variable	Suicidal ideation		Unadjusted		Adjusted	
	*n*	%	OR	95% CI	OR	95% CI
**Age**						
16–19	87	32.2	0.89	0.66–1.22	1.10	0.77–1.58
20–24	193	34.7	1	-	1	-
**Sex**						
Male	75	23.7	0.46	0.34–0.63**	0.54	0.39–0.77*
Female	205	40.3	1	-	1	-
**Experienced bullying**						
Yes	143	46.7	2.45	1.82–3.30**	1.89	1.35–2.65**
No	137	26.3	-	-	1	-
**Ever experienced unplanned pregnancy**						
Yes	37	45.7	1.74	1.09–2.76*	1.38	0.81–2.33
No	243	32.6	-	-	1	-
**Experienced education failure**						
Yes	105	39.5	1.44	1.06–1.94*	1.37	0.97–1.92
No	175	31.3	-	-	1	-
**Feel better off that friends**						
Yes	84	23.9	0.45	0.33–0.60**	0.64	0.45–0.90*
No	196	41.4	-	-	1	-
**Have underlying medical conditions**						
Yes	109	61.1	2.99	2.16–4.14**	2.12	1.47–3.05**
No	171	27.5	-	-	1	-
**Relationship conflicts**						
Yes	92	43.8	1.78	1.29–2.45**	1.24	0.85–1.80
No	188	30.5	1	-	1	-
**Body weight perception**						
Not normal	104	48.8	2.37	1.72–3.27**	1.65	1.15–2.37*
Normal	176	28.7	1	-	1	-
**Feeling about body appearance**						
Perfect	94	25.5	0.28	0.17–0.46**	0.46	0.26–0.79*
Moderate	140	37.4	0.49	0.31–0.80*	0.57	0.34–0.98*
Bad	46	54.8	1	-	1	-
**All needs met**						
Yes	101	27.2	0.58	0.43–0.78**	0.76	0.53–1.08
No	179	39.3	-	-	1	-
**Family financial support**						
Poor	92	43.6	1.86	1.29–2.70*	1.15	0.74–1.78
Moderate	102	31.7	1.12	0.79–1.57	0.96	0.65–1.42
Good	86	29.4	1	-	1	-
**Experienced sexual abuse**						
I am a survivor	33	62.3	4.23	2.36–7.56**	2.08	1.10–3.94*
Someone very close is	65	52.0	2.77	1.88–4.10**	1.79	1.15–2.78*
Never experienced sexual abuse	182	28.1	1	-	1	-

OR, odds ratio; CI, confidence interval.

* , *p* < 0.05;

** , *p* < 0.001.

Furthermore, having experienced bullying, unplanned pregnancy or failure in education, having underlying chronic medical conditions, having relationship conflicts, abnormal body weight perceptions, having poor family financial support and experience of sexual abuse or experience of sexual assault by/to someone very close were significantly associated with higher odds of having suicidal ideation. After adjusting for confounding variables, some of these factors (ever having experienced unplanned pregnancy or educational failure, having relationship conflicts, having all needs met and poor family financial support) were no longer significant.

### Gender variations in the factors associated with suicidal ideation (multiple logistic regression analysis)

In the crude multiple logistic model analysis (Table 3), experience of bullying, experience of educational failure, feeling better off than friends and someone very close having experienced sexual abuse were all significantly associated with suicidal ideation amongst male participants. However, after adjusting for covariates, all these factors became insignificant. Having underlying chronic illness and abnormal perception of body weight were associated with higher odds of suicidal ideation; having a moderate to perfect feeling about their appearance was associated with lower odds of suicidal ideation amongst the male participants. The magnitude and direction of association remained the same after adjustment in the logistic regression model analysis.

**TABLE 3:** Multiple logistic regression model analysis showing factors associated with suicidal ideation amongst males and females.

Variable	Males				Females			
	Unadjusted		Adjusted		Unadjusted		Adjusted	
	OR	95% CI	OR	95% CI	OR	95% CI	OR	95% CI
**Age**								
16–19	0.84	0.48–1.48	1.02	0.52–2.00	0.91	0.62–1.32	1.11	0.72–1.72
20–24	1	-	1	-	1	-	1	-
**Experienced bullying**								
Yes	2.50	1.47–4.24*	1.74	0.95–3.19	2.52	1.74–3.65**	2.01	1.32–3.06**
No	1	-	1	-	-	-	1	-
**Ever experienced unplanned pregnancy**								
Yes	1.08	0.41–2.83	0.88	0.28–2.70	1.91	1.09–3.32*	1.44	0.77–2.70
No	1	-	1	-	-	-	1	-
**Experienced educational failure**								
Yes	2.27	1.33–3.86*	1.79	0.96–3.33	1.19	0.81–1.74	1.20	0.79–1.84
No	1	-	1	-	1	-	1	
**Feel better off than friends**								
Yes	2.68	1.49–4.81**	1.00	0.54–1.85	3.01	2.02–4.51**	0.51	0.33–0.78*
No	1	-	1	-	1	-	1	-
**Have underlying medical conditions**								
Yes	2.68	1.49–4.81	2.24	1.11–4.51*	3.01	2.02–4.51***	2.14	1.37–3.33*
No	1	-	1	-	1	-	1	-
**Relationship conflicts**								
Yes	1.30	0.71–2.39	1.17	0.58–2.34	1.91	1.30–2.84	1.37	0.87–2.17
No	1	-	1	-	1	-	1	-
**Body weight perception**								
Not normal	2.76	1.56–4.89**	2.22	1.14–4.33*	2.13	1.43–3.15**	1.54	0.98–2.42
Normal	1	-	1	-	1	-	1	-
**Feeling about body appearance**								
Perfect	0.09	0.04–0.23**	0.10	0.04–0.28**	0.50	0.28–0.91*	0.90	0.46–1.77
Moderate	0.30	0.13–0.71*	0.30	0.11–0.77*	0.63	0.35–1.12	0.75	0.39–1.43
Bad	1	-	1	-	1	-	1	-
**All needs met**								
Yes	0.70	0.41–1.18	0.71	0.38–1.33	0.52	0.36–0.75**	0.69	0.45–1.08
No	1	-	1	-	-	-	1	-
**Family financial support**								
Poor	1.66	0.84–3.29	1.41	0.62–3.21	1.86	1.19–2.91*	1.04	0.61–1.79
Moderate	0.91	0.50–1.66	0.99	0.48–2.03	1.32	0.86–2.02	1.00	0.62–1.63
Good	-	-	1	-	1	-	1	-
**Experienced sexual abuse**								
I am a survivor	3.18	0.94–10.77	0.84	0.19–3.79	3.95	2.01–7.78**	2.50	1.20–5.21*
Some very close is a survivor	3.05	1.35–6.87*	2.12	0.82–5.52	2.33	1.48–3.66**	1.69	1.01–2.82*
Never experienced sexual abuse	1	-	1	-	1	-	1	-

OR, odds ratio; CI, confidence interval.

* , *p* < 0.05;

** , *p* < 0.01;

*** , *p* < 0.001.

Furthermore, the experience of being bullied, having underlying chronic illness, being a survivor of sexual abuse and having someone very close who had experienced sexual abuse were associated with higher odds of suicidal ideation amongst female participants. Although having poor family financial support, abnormal body weight perception, having experienced education failures, relationship conflicts and ever having experienced an unplanned pregnancy were associated with suicidal ideation amongst female participants, these factors all became insignificant after adjusting for other variables, using logistic regression model analysis.

## Discussion

Given the increasing trend of suicide and suicide attempts amongst students at HEIs of learning globally and in South Africa, this study sought to assess the rate of suicidal ideation, plans and influencing factors of suicidal ideation disaggregated by gender of students at an HEI in NMBM in South Africa. This study found a high prevalence of suicidal ideation (24.5%) amongst the students of this school. A previous South African study reported a similar prevalence of 25.4% amongst first-year university students.^24^ Other studies in Africa reported similar prevalence amongst college students (26.9% in Tunisia^25^ and 25.5% in Mozambique^26^). However, a lower prevalence of 13.5% was reported amongst college students in the United States of America.^27^ This finding was lower than the prevalence reported amongst Ethiopian university students, with lifetime prevalence of suicidal ideation and plans of 58.3% and 37.3%, respectively.^28^ The variations in the prevalence of suicidal ideation in different countries could be attributed to differences in sociocultural factors and the prevailing economic climate of those studies. In the present study, it should be noted that the students were experiencing a period of unrest (student protest) because of diminished financial support from their funders.^29^ Perhaps the financial hardship of the students contributed to the high prevalence observed in the study population.

The prevalence of suicidal plans was 9.6%, with females more likely to have suicidal plans than males. This finding was lower than the prevalence reported in another South African study, with a prevalence of suicidal plan of 12.6% in the preceding 12 months amongst the secondary school students.^30^ A plausible explanation for the observed lower prevalence of suicidal plans in our study in comparison to the study by Guedria-Tekari et al.^25^ could be the level of maturity or difference in age of the participants. In the current study, our participants were older and had attained post-secondary education, in comparison to the adolescents and high school students in the other study. More studies are needed in the sub-Saharan African region to elucidate the prevalence of suicidal plans amongst scholars. This information will provide insight for the Association of African Universities/Colleges on which to base regional policy on suicidal ideation and plans amongst students of higher learning.

Our study shows that more women than men reported lifetime suicidal ideations (30.1% vs. 15.5%). This finding corroborates previous reports from China^31^ and Mozambique.^32^ A possible explanation for this finding could be the higher occurrence of the significant risk factors of suicidal ideation such as experiences of sexual abuse, which are higher amongst female participants (8.3% vs. 3.5%). The psychological impact of sexual abuse is probably similar in both genders; however, the number of reported incidents of male sexual abuse experienced in our study was too small to make any significant deductions.

In this study, students who had experienced bullying, especially female students, had higher odds of suicidal ideation. These findings are in agreement with previous studies, which reported victims of bullying being at higher risk of suicidal behaviour.^18,26,33^ The increased suicide risk may be attributed to untoward sequelae of bullying, which include depression, anxiety, substance abuse and the feeling of not belonging.^26^ School authorities could potentially screen for experiences of bullying and its behavioural complications in order to mitigate suicidal ideation at HEIs.

This study also found that having underlying chronic illnesses was significantly associated with suicidal ideation, with a higher risk amongst those with two or more chronic diseases. This finding is consistent with the results of previous studies.^34,35^ A Canadian study reported a higher prevalence of suicidal ideation amongst people with chronic diseases, and the risk increased with the number of comorbidities.^34^ Similarly, Ferro et al.^35^ reported a higher risk of suicidal ideations amongst patients with mental illness. It is plausible that many students did not have access to clinical support and care. This is evidenced by the fact that there are shortages of psychologists on many of the college campuses. Similarly, the shortages of psychologists are a critical challenge within the public sector’s healthcare facilities. This finding has significant implications for planning of interventions directed toward managing underlying chronic illnesses, including mental illness, amongst students of HEIs in the region. Furthermore, this finding highlights the urgency of incorporating screening for chronic illnesses, including mental illness and suicidal ideation, during school registrations.

Given the high HIV prevalence (7.9%)^32^ in the age group of 15–24 years, included in this study, it should be noted that only 25 students reported a diagnosis of HIV in this cohort (3%). Hence, this finding should be treated with caution because of the possibility of under-reporting of HIV status by the participants. Sexual assault increased the risk of suicidal ideation, whether through personal experience or that of someone very close to them. Some previous studies have reported similar findings.^36,37^ The increased odds of suicidal ideation amongst rape survivors have been attributed to the cascade of emotional response that follows the traumatic experience, from self-blame to depression and post-traumatic stress disorder.^6^

Participants’ perception of abnormal body weight was associated with increased odds of suicidal ideation. This is more true for males compared to females. Similarly, participants who felt that their body appearance was moderate to perfect had lower odds of suicidal ideation, especially amongst the male students. This finding is consistent with results from previous studies that reported that underweight males and males with normal weight but body weight dissatisfaction were most vulnerable to suicidal ideation.^38,39^ Similarly, a study conducted amongst adolescents in China showed that only males with the perception of underweight or obesity were at higher risk of suicidal ideation.^39^ On the contrary, Shin et al.^40^ in Korea reported that only women with distorted perceptions of body weight were more likely to express suicidal ideation than those who perceived a normal body image. The present study highlights the need to pay attention to body perceptions amongst young male students with a view to promptly recognising suicidal tendencies in them.

In our study, cannabis and alcohol usage in the preceding 12 months did not show any significant association with having suicidal ideations. This finding is contrary to what has been reported in previous studies, in which alcohol and cannabis use were significantly associated with suicidal ideation.^41,42,43^ It should be noted that the participants included in our study were younger (18–24 years) in comparison to other studies (ages up to 60 years) that reported this association.^41,42,43^. Furthermore, we did not quantify the intensity of alcohol use in our study.

## Strength and limitations

This is the first study to report the prevalence of suicidal ideation and plans in any HEI in the Eastern Cape Province, South Africa. In addition, this study adopted multistage cluster random sampling of a representative (large) sample of students in the selected institution. This study therefore provides a reliable data baseline for the school authority, the Department of Higher Education and other relevant stakeholders in the province and country to craft policy on screening for suicidal ideation.

However, the limitations of the study cannot be ignored. The cross-sectional nature of the study precludes causal association between the risk factors and suicidal ideation. Self-reported lifestyle behaviours and underlying mental and chronic medical conditions may have been under-reported. Although the investigators anonymised the data collection instrument in order to limit the effect of social desirability bias, under-reporting of medical and mental illnesses may have occurred. Lastly, we did not capture the anthropometric measurements of the participants to determine their body mass index; therefore, we could not draw any conclusions as to whether their perception of their body weight was an accurate or distorted concern.

## Conclusion

We report a high prevalence of suicidal ideations and plans amongst students of higher education in the NMBM, Eastern Cape, with significant gender variations in the influencing factors. The study further demonstrated a significant association between suicidal ideation and gender, bullying experience, having underlying mental illness, having had a sexual assault experience and perception of body image. The various stakeholders of the college should consider implementing a screening strategy towards identifying the risk factors as well as suicidal ideation, with prompt interventions for the students.
